# Implementation of third-generation digital PCR for non-invasive prenatal diagnosis of sickle cell disease and early detection. Pilot study

**DOI:** 10.3389/fmed.2026.1815629

**Published:** 2026-05-08

**Authors:** Sara Ferrer Benito, Miguel Gómez Álvarez, María Josefa Torrejón Martínez, Montserrat López Rubio, Rafael Andrés del Orbe Barreto, Jaime Arbeteta Juanis, María Josefa Muruzabal Siges, Eduardo José Salido Fiérrez, Aránzazu García Mateo, Valle Recasens, Jorge Martínez Nieto, Ana Villegas Martínez, Fernando Ataúlfo González Fernández, Ana Belén Ortega Montero, Laura García Moreno, Paloma Ropero Gradilla, Celina Benavente Cuesta, Spanish Group of Erythropathology

**Affiliations:** 1 Hematology and Hemotherapy Service, Hospital Clínico San Carlos, Madrid, Spain; 2 Sanitary Research Institute HCSC, Madrid, Spain; 3 Clinical Analysis Service, Hospital ClSan Carlos, Madrid, Spain; 4 Hematology and Hemotherapy Service, Hospital Universitario PrFelipe, Alcal1 de Henares (Madrid), Madrid, Spain; 5 Hematology and Hemotherapy Service, Hospital Universitario de Cruces, Bilbao, Spain; 6 Hematology and Hemotherapy Service, Hospital Universitario de Guadalajara, Guadalajara, Spain; 7 Hematology and Hemotherapy Service, Hospital Universitario Marqu9s de Valdecilla, Santander, Spain; 8 Hematology and Hemotherapy Service, Hospital ClUniversitario Virgen de la Arrixaca, Murcia, Spain; 9 Hematology and Hemotherapy Service, Hospital General de Segovia, Segovia, Spain; 10 Hematology and Hemotherapy Service, Hospital Universitario Miguel Servet, Zaragoza, Spain

**Keywords:** cell-free fetal DNA (cffDNA), digital PCR (dPCR), hemoglobin S (HbS), minimally invasive prenatal diagnosis (NIPD), sickle cell disease (SCD)

## Abstract

Sickle cell disease (SCD) is one of the most prevalent autosomal recessive conditions worldwide, affecting more than 600,000 newborns annually. Despite advances in treatment, it remains a chronic condition requiring lifelong management. Allogeneic transplantation and gene therapy are currently the only curative options when applicable and available. We present the results of a pilot study evaluating the use of digital PCR (dPCR) for non-invasive prenatal diagnosis (NIPD), a minimally invasive approach for SCD, based on relative pathogenic variant dosage (RMD, Relative Mutation Dosage) analysis in maternal plasma. Nine pregnant women carrying the pathogenic variant (βA/βS) were included. RMD values ranged from 0.436 to 0.501 (mean: 0.475) in cases with heterozygous fetuses and from 0.377 to 0.431 (mean: 0.397) in cases with wild-type fetuses. Z-score classification correctly identified fetal genotype in all conclusive cases (8/9), with one inconclusive result. Results were confirmed postnatally with 100% concordance in classified cases. These findings support the feasibility of dPCR-based NIPD for SCD as a minimally invasive approach. However, further validation in larger cohorts and with comprehensive quality control parameters is required.

## Introduction

Sickle Cell Disease (SCD) is a chronic condition characterized by moderate to severe hemolytic anemia that usually manifests from the third or fourth month of life. It is accompanied by vasculopathy, leading to vaso-occlusive events that cause ischemia and chronic damage in multiple organs. The disease is interrupted by acute episodes, and patients are highly susceptible to infections due to the lack of splenic function, massive hemolytic events, and the risk of acute chest syndrome, which can result in premature death (1).

SCD is caused by a single-nucleotide pathogenic variant in codon 6 (GAG>GTG) of the β-globin gene (HBB: c.20A > T; NM_000518.4). This pathogenic variant leads to the production of hemoglobin S (HbS), characterized by the substitution of glutamic acid for valine at position β6 (A3) (2). This change allows HbS to form polymers in the deoxygenated state, distorting red blood cell morphology into the characteristic sickle shape, thereby obstructing microvascular circulation and triggering clinical symptoms.

About 7% of the global population is a carrier of an abnormal hemoglobin gene, with over 600,000 children affected by SCD each year. The vast majority of SCD cases (over 83%) derive from the high prevalence of the sickle cell gene in specific regions such as the Middle East, South Asia, the Mediterranean, and sub-Saharan Africa. Population migration and interethnic marriages have introduced SCD into nearly every country in the world, including non-endemic areas like northern Europe (3).

In Spain, according to REHem data, there are 1,142 registered cases, with a steady increase driven by migration from the Caribbean, South America, and Africa. Early detection, prophylactic penicillin treatment, and vaccines have improved the patients' quality of life and life expectancy. Despite that, SCD remains a debilitating condition requiring lifelong attention; allogeneic transplantation is the only curative option if an HLA-compatible donor exists (4).

Prenatal diagnosis offers pregnant couples the possibility of rapid, accurate information about the fetus's genetic health. This enables informed decision-making and allows appropriate emotional and medical preparation. Although conventional techniques such as chorionic villus sampling (CVS) and amniocentesis are effective, these are invasive procedures that carry a small procedure-related risk of miscarriage, largely dependent on operator experience (5–8).

To address these challenges, Non-invasive Prenatal Diagnosis (NIPD) has been developed, based on cell-free fetal DNA (cffDNA) present in maternal blood from placental trophoblastic cells (9–11). Fetal DNA concentration varies throughout pregnancy and in response to maternal and fetal factors. In the past two decades, there has been significant progress in understanding cffDNA, since its presence allows obtaining fetal genetic information from maternal blood (12, 13).

Despite these advances, NIPD faces difficulties due to the coexistence of maternal and fetal DNA, and the low concentration of cffDNA in maternal plasma, which has led to false negatives and incorrect diagnoses in some cases. Nevertheless, NIPD has become integral to clinical practice, being used to determine fetal sex (14), assess RhD status (15), screen for common aneuploidies, and diagnose paternal or *de novo* pathogenic variants (16).

For monogenic diseases, NIPD application has been limited to paternal and *de novo* inheritance due to the complexity of distinguishing maternal from fetal alleles. In these cases, analytical methods vary depending on the underlying genetic basis, including quantitative fluorescent PCR (QF-PCR), real-time PCR, and minisequencing or SNaPshot (17).

In cases where both parents carry the same pathogenic variant, NIPD cannot rely on simple pathogenic variant detection due to the coexistence of maternal and fetal DNA. Instead, it is based on the analysis of relative pathogenic variant dosage (RMD, Relative Mutation Dosage) using digital PCR (dPCR), which enables the detection of subtle imbalances between wild-type and variant alleles (13–17). dPCR allows absolute quantification of wild-type and variant alleles with high sensitivity and specificity (18). This quantification enables identification of allelic balance or imbalance between normal and variant alleles in the specimen: in a non-transfused, non-pregnant woman, allelic equilibrium (50:50 between wild-type and variant alleles) is expected. The introduction of fetal DNA into maternal plasma can produce an imbalance: if the mother and fetus share the same genotype, balance is maintained; whereas if the fetus is homozygous, there will be an increase in whichever allele the fetus is homozygous for (19).

We present preliminary results of a pilot study in the process of validating an NIPD method for SCD detection using dPCR.

## Materials and methods

An observational study with diagnostic intervention included nine pregnant women with sickle cell trait (βA/βS) and non-pregnant women with all three possible genotypes: six wild-type homozygotes (βA/βA), eight heterozygotes (βA/βS), and six pathogenic variant homozygotes (βS/βS).

Women who had recently received blood transfusions, allogeneic transplants, immunological or stem cell therapies, or had multiple gestations were excluded due to possible interference from exogenous DNA.

Samples were collected from several hospitals in Madrid (Hospital Clínico San Carlos, Universitario de Getafe, Universitario Príncipe de Asturias) and from hospitals in Bilbao (Universitario de Cruces), Guadalajara (Universitario de Guadalajara), Santander (Universitario Marqués de Valdecilla), Murcia (Clínico Universitario Virgen de la Arrixaca), Segovia (General de Segovia), and Zaragoza (Universitario Miguel Servet).

Samples were processed and molecularly characterized in the Erythropathology laboratory at the Hematology Service of Hospital Clínico San Carlos. Conventional hematimetric study and reticulocyte counts were performed using an automatic cell counter (UniCel DxH 800, Coulter Cellular Analysis System, Beckman Coulter). Identification and quantification of HbS and other hemoglobin fractions were carried out using ionic exchange HPLC (Variant II, BioRad) and capillary electrophoresis (Minicap Flex Piercing, Sebia).

For molecular analysis, peripheral blood DNA was extracted automatically from leukocytes (EZ1, Qiagen) and characterized using the commercial β-Globin StripAssay^®^ MED kit (ViennLab). Collection of circulating free DNA used maternal peripheral blood between gestational weeks 10 and 20 in K3EDTA Cell-free DNA BCT tubes (Streck). Extraction of circulating free DNA was achieved using the commercial QIAamp Circulating Nucleic Acid kit (Qiagen).

Molecular characterization of circulating free DNA was performed by dPCR [QuantStudio 3D Digital PCR System (Thermo Fisher)] using TaqMan-specific probes for the variant allele (FAM) and the wild-type allele (VIC) (Table 1). This enabled absolute quantification of both alleles via QuantStudio 3D AnalysisSuite Cloud Software (ThermoFisher) and calculation of the RMD for the pathogenic variant (HBB: c.20A > T) using the following formula:


$$\begin{array}{l} \text{RMD}=\left[\text{Number of FAM}-\text{positive events }\left(\text{variant}\right)\right]\\ \;/\left(\text{Number of total VIC}+\text{ FAM}-\text{positive events}\right). \end{array}$$

The obtained RMD values were translated into z-score values using a formula (20) based on a reference population of non-pregnant heterozygous women, establishing equilibrium/imbalance ranges to associate a particular fetal genotype.


$$\begin{array}{l} z-score=\left(x-\text{μ}\right)/\text{σ} \end{array}$$

where:

x = obtained RMD

μ = mean of the reference population

σ = standard deviation of the reference population

**Table 1:** Sequences of primers and probes used in digital PCR.

Oligonucleotide	Sequence (5^′^-3^′^)
Forward primer	CCCCACAGGGCAGTAACG
Reverse primer	AGCAACCTCAAACAGACACCAT
VIC assay probe	CTGACTCCTGAGGAGAA
FAM assay probe	CTGACTCCTGTGGAGAA

The nucleotide that differentiates the wild-type sequence from the mutated sequence is shown in red.

The probe labeled with the VIC fluorophore detects the wild-type allele, whereas the probe labeled with the FAM fluorophore detects the hemoglobin S pathogenic variant.

Cut-off points were determined to classify fetal genotypes with 99% confidence: z-score values less than −2.6 were associated with pregnant women carrying wild-type homozygote fetuses; those greater than +2.6 indicated pregnant women with homozygous affected fetuses. Values between −1.5 and +1.5 were assigned to pregnant women with heterozygous fetuses, with an intermediate zone where results were considered inconclusive.

Once a diagnosis in pregnant women was achieved, the results were verified. Neonatal diagnosis was performed via universal heel-prick screening for hemoglobinopathies.

For descriptive statistical analysis, qualitative variables are presented with their frequency distribution. Quantitative variables are summarized with their mean and standard deviation (SD). Where quantitative variables are asymmetrically distributed, median and interquartile range (IQR) are reported. The z-score was used to classify patients in the group of pregnant carriers of HbS.

Data processing was performed using IBM SPSS Statistics v.26 and Microsoft Excel.

The study was conducted with informed consent from all participants and was approved by the CEIm Hospital Clínico San Carlos of Madrid.

## Results

### RMD analysis using digital PCR

**Figure 1:**
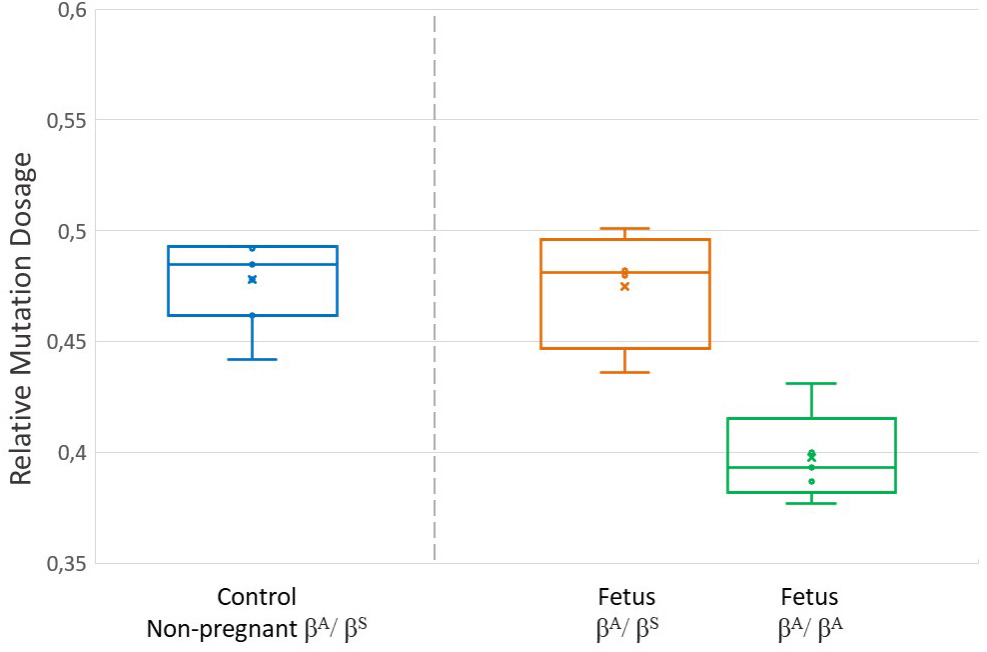
RMD values obtained by digital PCR. The graph shows the RMD values in pregnant women grouped according to the fetal genotype (orange = βA/βS pregnant women carrying a βA/βS fetus; green = βA/βS pregnant women carrying a βA/βA fetus). RMD values obtained in the control group are also shown (blue = non-pregnant βA/βS women). (*n* = 9 pregnant women).

dPCR was used to determine the RMD of variant alleles in the different study groups. RMD values showed clear differentiation among non-pregnant women with various genotypes for the pathogenic variant HBB: c.20A > T. Homozygous pathogenic variant carriers had RMD values close to 1 (mean: 0.96), wild-type homozygotes had values near 0 (mean: 0.02), and heterozygotes maintained allelic balance, with values from 0.442 to 0.493 (mean: 0.478) (Figure 1, Table 2).

**Table 2:** RMD values obtained in each control group of non-pregnant women.

Genotype (non- pregnant women)	Mean	Minimum value	Maximum value
β^A^/β^A^ (*n* = 6)	0.026	0.004	0.058
β^A^/β^S^ (*n* = 7)	0.478	0.442	0.493
β^S^/β^S^ (*n* = 6)	0.960	0.934	0.998

**Figure 2:**
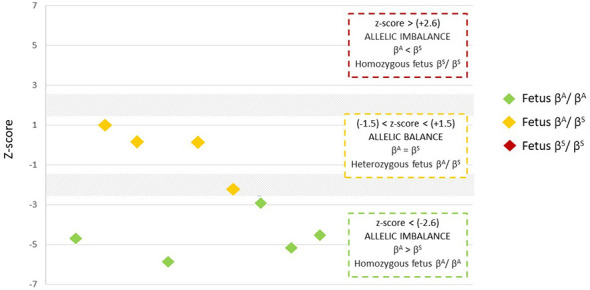
z-score values derived from RMD measurements. The three regions defining the fetal genotype are shown, along with two areas of uncertainty (gray zone). Each value is represented by a diamond, and the color is determined according to the fetal genotype confirmed after birth (orange = βA/βS; green = βA/βA). (*n* = 9 pregnant women).

In the group of pregnant women, all carriers of SCD, distinctive results were observed depending on fetal genotype. Results showed variations in RMD values that allowed effective differentiation between heterozygous and wild-type fetuses. Specifically, pregnant women with heterozygous fetuses had RMD values ranging from 0.436 to 0.501 (mean: 0.475), whereas those with wild-type fetuses ranged from 0.377 to 0.431 (mean: 0.397) (Figure 2, Table 3).

**Table 3:** RMD and z-score values were obtained, and their interpretation in each pregnant woman included in the study.

Pregnant woman βA/βS (*n* = 9)	RMD	z-score	Fetal genotype obtained	Postnatal genotype
1	0.393	−4.78	β^A^/β^A^	β^A^/β^A^
2	0.501	1.27	β^A^/β^S^	β^A^/β^S^
3	0.482	0.17	β^A^/β^S^	β^A^/β^S^
4	0.377	−5.93	β^A^/β^A^	β^A^/β^A^
5	0.480	0.1	β^A^/β^S^	β^A^/β^S^
6	0.436	−2.36	Indeterminate	β^A^/β^S^
7	0.431	−2.66	β^A^/β^A^	β^A^/β^A^
8	0.387	−5.18	β^A^/β^A^	β^A^/β^A^
9	0.4	−4.62	β^A^/β^A^	β^A^/β^A^

The table shows the RMD and z-score values obtained for each case, and the corresponding fetal genotype determined using this technique. The actual fetal genotype confirmed after birth is also presented. Gestational age and fetal fraction were not available in the original study design.

### Fetal Genotype determination by z-score

Genetic characterization revealed an interesting distribution of obtained values. Five cases fell within the green zone with z-score values between −5.93 and −2.66 on the reference scale, indicating a specific range of values that may correspond with normal or balanced parameters in the analyzed genetic context. Three results were in the yellow zone, with z-scores from 1.27 to 0.1, possibly suggesting some degree of imbalance relative to the reference standard. One result was in the gray zone, with a value of −2.36, which may indicate an intermediate case needing further analysis due to its distance from conventional reference ranges.

These findings are depicted in Figure 2, situating the z-score values for each result. This graphical representation aids understanding and visualization of the distribution of results in each zone, enabling better data interpretation.

### Fetal genotype verification and confirmation

After applying cut-off points, fetal genotype was verified by alternative diagnostic methods, including neonatal screening for hemoglobinopathies. Results were 100% concordant for wild-type homozygote and heterozygote fetuses. However, one case in the intermediate zone was inconclusive and required repeat testing, corresponding to a pregnant woman with a heterozygous fetus.

Among heterozygous pregnant women carrying heterozygous fetuses, z-score classification indicated allelic equilibrium in 3 out of 4 cases. In contrast, among heterozygous pregnant women carrying wild-type fetuses, 4 out of 5 cases were correctly classified as imbalanced.

Given the small sample size, these results should be interpreted with caution and do not allow robust estimation of diagnostic performance.

To date, no cases have included pregnant women carrying homozygous affected fetuses.

## Discussion

Monogenic recessive diseases have not previously benefited from NIPD due to the inability to separate maternal and fetal DNA, posing a challenge for the detection of maternally inherited variants (21).

This study highlights dPCR as an optimal and valuable technique for NIPD of SCD in mothers who are pathogenic variant carriers (22). RMD analysis by dPCR revealed distinctive patterns among non-pregnant heterozygous and homozygous pathogenic variant carriers. In the pregnant group, analysis was based on RMD and fetal contribution. Allelic balance is expected in cases where the mother and fetus are both heterozygous, with a slight imbalance observed in wild-type homozygote fetuses (13, 19).

In nine cases of pregnant women with the SCD pathogenic variant, variations in RMD were observed depending on fetal genotype and the amount of fetal DNA in maternal plasma (13), allowing effective differentiation between heterozygous and wild-type homozygote fetuses.

Values similar to controls were observed in pregnant women with heterozygous fetuses (control mean: 0.478; mean for pregnant women with heterozygous fetuses: 0.475), indicating maintained allelic balance. Conversely, pregnant women with wild-type fetuses had values deviating from balance (mean: 0.397), attributable to fetal DNA increasing wild-type allele copies and decreasing RMD. Although no mutant homozygote fetus cases were found in this study, the opposite could be expected for such cases, with an increase in mutant allele copies and RMD values near 1. Thus, relative pathogenic variant dosage varies by fetal genotype due to fetal DNA in maternal blood, confirming findings by Perlado and Hanson for NIPD of recessive monogenic disorders (19, 24).

RMD alone provides a distinctive pattern; due to low fetal DNA concentrations, z-score calculation is needed to differentiate fetal genotypes. This measure, comparing the RMD of each pregnant woman to controls, determines fetal genotype. In our study, all fetuses with z-score > −2.6 were correctly classified as wild-type homozygotes (5/5); fetuses with values between 1.3 and 0.1 were correctly classified as heterozygotes (3/4); only one remained inconclusive in the intermediate “gray zone,” which adds safety by avoiding false positives/negatives (19, 23).

Results from dPCR genotyping in pregnant pathogenic variant carriers are promising for NIPD and align with previous research on non-invasive diagnosis of monogenic disorders (25).

These findings should be interpreted in the context of previous studies, such as Shaw et al. (26), which highlight the importance of strict quality control parameters and adequate fetal fraction thresholds in ensuring robust NIPD performance.

The precision and differentiation capability observed are consistent with key features of dPCR-based RMD analysis, supporting its feasibility for fetal genotyping in pregnant pathogenic variant carriers within a pilot study context. These preliminary findings support promising analytical sensitivity and specificity of dPCR for detecting variant alleles, and suggest its potential as a safer, less invasive alternative for prenatal diagnosis of SCD, within the limitations of the current pilot design (24).

Several limitations must be acknowledged. First, the sample size is small, limiting statistical power and generalizability. Second, no cases involving pregnant women carrying homozygous affected fetuses were included, which restricts the assessment of the full diagnostic capability of the method.

The assay's performance should be interpreted as a test of methodological feasibility. Key metrics, such as fetal fraction estimation and comprehensive quality control parameters for circulating cell-free DNA (cfDNA), were not part of the initial study design, meaning this study does not represent a final analytical benchmark.

Furthermore, the reference population used for z-score calculation was limited, which may affect classification robustness.

Clinically, these findings have important implications: the ability to determine fetal genotype minimally invasively can enable improved parental decision-making and clinical care planning. This technique might also reduce the risks linked to invasive prenatal procedures, thus improving maternal and fetal safety (5–8).

The success of this research highlights the importance of continued development of NIPD tools. The effective application of dPCR for fetal genotyping in SCD suggests its potential usefulness for other hereditary monogenic diseases, paving the way for future research exploring its utility in a broader range of genetic conditions.

Taken together, these results show the feasibility of dPCR-based NIPD for sickle cell disease in a pilot setting. However, while these findings are promising, further research with larger sample sizes, explicit fetal fraction assessment, and standardized quality control metrics remains essential to fully validate its clinical implementation and explore its broader diagnostic potential for other genetic disorders.

## Author's note

This manuscript is a translated version of a previously published work. The original article was published as: Ropero P, Ferrer S, Gómez M, et al. Implementación de la PCR digital para el diagnóstico prenatal no invasivo de la enfermedad de células falciformes. Estudio piloto. An RANM. 2024;141(01): 19-27. DOI: http://dx.doi.org/10.32440/ar.2024.141.01.rev02. The content has been adapted to comply with Frontiers' guidelines, and wording has been revised to avoid any implication of novel research.

## Data Availability

The original contributions presented in the study are included in the article/supplementary material, further inquiries can be directed to the corresponding author.
